# Development of a simple and effective online training for health workers: results from a pilot in Nigeria

**DOI:** 10.1186/s12889-022-12943-1

**Published:** 2022-03-21

**Authors:** Marshall P. Thomas, Samantha Kozikott, Moreen Kamateeka, Ramatu Abdu-Aguye, Emmanuel Agogo, Bakunawa Garba Bello, Karen Brudney, Olivier Manzi, Leena N. Patel, Amy Elizabeth Barrera-Cancedda, Jobin Abraham, Christopher T. Lee

**Affiliations:** 1 Resolve to Save Lives, an Initiative of Vital Strategies, New York, NY USA; 2 African Field Epidemiology Network (AFENET), Abuja, Nigeria; 3 National Primary Health Care Development Agency (NPHCDA), Abuja, Nigeria

**Keywords:** Online learning, Mobile health, Primary health care, Health workers, Infection prevention and control, Nigeria, Africa, Design thinking, Learning science

## Abstract

**Background:**

Health workers (HWs) in Africa face challenges accessing and learning from existing online training opportunities. To address these challenges, we developed a modular, self-paced, mobile-ready and work-relevant online course covering foundational infection prevention and control (IPC) concepts. Here, we evaluate the first pilot of this course, conducted with HWs in Nigeria.

**Methods:**

We used a learner-centered design and prototyping process to create a new approach to delivering online training for HWs. The resulting course comprised 10 self-paced modules optimized for use on mobile devices. Modules presented IPC vignettes in which learning was driven by short assessment questions with feedback. Learners were recruited by distributing a link to the training through Nigeria-based email lists, WhatsApp groups and similar networks of HWs, managers and allied professionals. The course was open to learners for 8 weeks. We tracked question responses and time on task with platform analytics and assessed learning gains with pre- and post-testing. Significance was evaluated with the Wilcoxon signed-rank test, and effect size was calculated using Cohen’s *d*.

**Results:**

Three hundred seventy-two learners, with roles across the health system, enrolled in the training; 59% completed all 10 modules and earned a certificate. Baseline knowledge of foundational IPC concepts was low, as measured by pre-test scores (29%). Post-test scores were significantly higher at 54% (effect size 1.22, 95% confidence interval 1.00-1.44). Learning gains were significant both among learners with low pre-test scores and among those who scored higher on the pre-test. We used the Net Promoter Score (NPS), a common user experience metric, to evaluate the training. The NPS was + 62, which is slightly higher than published scores of other self-paced online learning experiences.

**Conclusions:**

High completion rates, significant learning gains and positive feedback indicate that self-paced, mobile-ready training that emphasizes short, low-stakes assessment questions can be an effective, scalable way to train HWs who choose to enroll. Low pre-test scores suggest that there are gaps in IPC knowledge among this learner population.

## Background

### Infection prevention and control

There is increasing recognition of the importance of infection prevention and control (IPC) as a key component of epidemic preparedness and response and as a critical tool for keeping health workers (HWs) safe [1–3]. HWs have higher rates of infection than the general population, particularly during epidemics, and weak IPC programs as well as gaps in emergency preparedness contribute to this risk [1]. The COVID-19 pandemic has further highlighted the association between IPC and HW safety [4, 5]. In the early months of the pandemic, HWs were three times more likely than members of the general population to have COVID-19 [6]. While effective strategies for improving IPC programs and practices are an active area of research, an emerging consensus indicates that multiple synergizing approaches, including training, are required to bring about improvements in IPC [7–9]. Notably, HWs who received IPC training were at lower risk of developing COVID-19 [6]. Effective and continuous training is particularly important to keep HWs updated on rapidly evolving IPC guidance in the context of COVID-19 [10]. Ongoing training will also be needed to address new epidemics of other infectious agents.

The World Health Organization (WHO) defines HWs broadly to encompass health service providers as well as managers and support personnel [11]. A wide array of different HWs (including doctors, nurses, administrators, laboratory staff and community health workers) play a role in IPC implementation and require some foundational IPC knowledge to do their jobs safely and effectively [7]. In addition, officials and HWs at all levels of the health system (national, subnational, district and local) play a role in IPC. However, IPC training is far from universal. A recent assessment of 88 countries’ national IPC programs found that just over half provide in-service IPC training, while fewer than half provide pre-service IPC training [12]. In a recent survey comparing HWs’ access to personnel trained in IPC across the six different WHO regions, fewer than 40% of respondents in the African region reported that they had access to trained personnel “always” or “often,” the lowest of any WHO region [13]. Although such surveys indicate that many HWs do not currently receive IPC training, less is known about the IPC knowledge of individual HWs across different countries and in different positions in the health system. Integrating assessments into training may be helpful to better define gaps in IPC knowledge and skills and develop targeted interventions to address those gaps.

### Online learning: limitations and solutions

Many organizations replaced in-person training with online learning in response to restrictions on gathering and travel imposed during the COVID-19 pandemic. This shift required an expansion of online learning opportunities for HWs, which currently include massive open online courses (MOOCs) [14], webinars [15] and synchronous virtual communities of practice [16]. However, accessing, engaging with and learning in online environments can be challenging even under optimal circumstances. Though MOOCs and other online courses are lauded for their potential to deliver learning at scale, their completion rates are low [17, 18]. Moreover, high rates of learner multitasking online can impede focused learning [19]. While these findings hint at the impediments to achieving depth of impact and scale through online learning [17], a growing body of research points to best practices in online course design that improve engagement and learning. Simple and organized online learning experiences lead to less frustration and sustain learners’ sense of self-efficacy [20], while focusing on only essential content improves knowledge retention [21]. Assessment that is designed to provide feedback and promote learning rather than to evaluate learners (formative assessment) is a powerful way to activate learning offline and online [22, 23]. The addition of short explanations after assessment questions improves learners’ perceptions of the learning experience [24]. Course design factors also influence learner engagement and retention. For example, shorter online courses and those with deadlines have higher completion rates [25, 26].

### Online learning for HWs in Africa

In both online and face-to-face learning, content- and learner-specific factors influence course completion and learning outcomes. When learners believe content has career utility or relevance to their daily professional lives, they are more likely to persist [27] and HWs report higher interest in online learning that is relevant to their work [28]. The efficacy of different online learning approaches is context-dependent: working adults who prioritize flexibility prefer self-paced learning, but this modality might disadvantage learners who are less motivated or need more structure. Moreover, online learning is not equally suited to all of the knowledge, skills and abilities that HWs need to develop [29]. It can be challenging to teach and assess complex clinical skills in a virtual setting [28]. Online learning may be a useful tool to address existing gaps in IPC training, but more work needs to be done to delineate what IPC knowledge, skills and behaviors can be effectively taught online.

Residents of low- and middle-income countries face some additional barriers to learning online. In Africa, most internet users connect using mobile broadband (rather than fixed connections), and mobile network coverage is still rapidly growing. As of 2020, an estimated 77% of the African population is within range of a 3G wireless signal [30], though it is likely that access to internet and mobile devices is higher among HWs [13]. Factors other than the reach and quality of internet connections (including reliability of electrical service, wireless data and device costs and digital literacy skills gaps) represent additional barriers to access and use of internet resources [30, 31]. This environment favors the adoption of simple, mobile-compatible and user-friendly web applications that do not consume much data.

Although the objective of most training is to develop learners’ knowledge and skills, more accessible metrics, such as participation, completion and learner satisfaction are more frequently evaluated [32]. Only a fraction of studies evaluating online medical education in low- and middle-income countries report learning gains [33]. There are comparatively few studies that assess fully online training of in-service HWs in Africa, perhaps in part because the continent is under-represented in the medical education research literature [34]. In the case of one app-based training for nurses and midwives in Rwanda, learners received a half-day in-person introduction to the app and were coached on its use during frequent site visits [35]. In a training on Ebola Virus Disease in Nigeria, HWs accessed the training via tablets placed in health facilities [36]. There were significant short-term learning gains in both studies, though long-term knowledge gains were not assessed. It may be logistically complex and costly to scale up interventions that involve providing learners with devices and in-person support, challenges that are heightened by COVID-19-related travel restrictions and safety measures. These cases highlight the need for more research on the efficacy and limitations of fully online training for learners in Africa.

In this work, we hypothesized that online learning for HWs in Africa could be improved by designing learning experiences that play to the strengths of online learning and proactively address the barriers faced by this population of learners.

## Methods

### Training development process

We used a learner-centered design thinking process [37] to develop this training. Our process involved understanding learners’ needs and the challenges they face, defining solutions that would address those needs (Table 1), and developing and iteratively testing solutions. Through our experiences delivering a large-scale HW training program during the COVID-19 pandemic [1], conversations with colleagues and health workers in Africa and our own experiences with online learning, we developed features to address the learning needs of HWs in Africa, with a focus on workers in primary health care facilities. We took a broad view of needs to encompass the myriad challenges that learners might face in accessing online learning, remaining engaged and retaining knowledge.

**Table 1:** Features that address the learning needs of HWs in Africa

Learners’ context and challenges	Features to include in learning solutions
**Mobile devices and connectivity**: most users access internet with mobile devices using cellular data	**Mobile-ready and low bandwidth**: optimized for mobile devices and for use over 3G connections
**Lengthy registration processes**: some platforms have multi-step registration processes or require download of an app	**Minimal barriers to entry**: does not require the download of new software, has a straightforward registration process and is offered free of charge
**Complex user interfaces**: some platforms have many different menus, screens, and pages that may have limited utility for learners and can interfere with learning	**User-friendly and simple**: has a single-screen and single-button navigation experience that does not require any orientation for learners to get started and is focused on learners’ priorities and experience
**Scheduling issues**: HWs’ work may conflict with synchronous events (webinars)	**Self-paced and brief:** can be accessed at any time and broken into short modules
**Lack of utility**: many courses focus on practices appropriate for tertiary care settings or emphasize non-clinical basic science content	**Focused and applicable**: learning is driven by brief “vignettes” set in health care facilities, content focuses on the most fundamental concepts in IPC, and certificates emphasize job-related course content
**Passive learning**: online courses often rely heavily on video and text, with limited opportunities for interactivity	**Immersive learning**: learning begins as quickly as possible and is driven by feedback and brief explanations embedded in formative assessment

We developed a prototype that met these design parameters and shared this module and written descriptions of the overall training structure with team members, colleagues and HWs in Nigeria. We then adapted the prototype based on their feedback. Notably, we changed the assessments to give learners more control over their progress and adjusted the target word count of the modules based on the time it took these users to complete them. We also changed the way we released content to learners. Our initial design implemented spaced repetition [38], with modules released on a schedule. Based on comments received in the prototyping process, we removed this constraint to allow learners to access any module at any time.

**Fig. 1:**
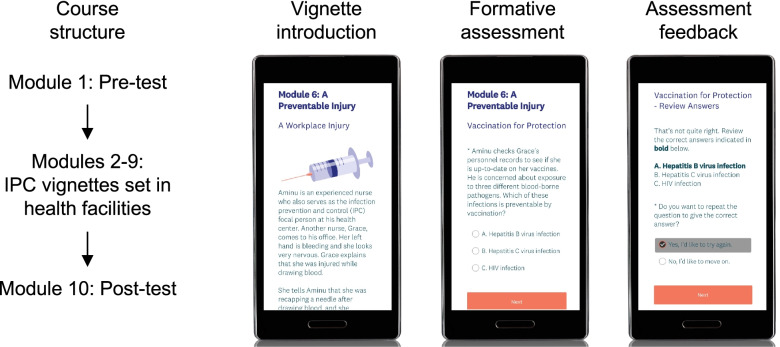
Course structure and screenshots of the prototype. Learners could access the modules in any order, though linear progression was encouraged. Screenshots are shown in mobile view, but all modules were also compatible with computers and tablets

The final prototype (Fig. 1) comprised 10 modules with each module designed to require 10 to 20 min of work. All modules and supporting materials were in English. The first and last modules were the pre-test and post-test, respectively; these tests had no time limit and contained the same questions. The intervening modules each presented a brief vignette, occurring in a primary health care facility, in which learners answered questions and received feedback as a means of learning about IPC concepts in an interactive and narrative fashion. We did not find an existing learning management system suitable for this prototype, so we adapted other platforms to fit our purposes. We used a web-based survey tool (SurveyMonkey) to deliver the modules and an enterprise email service (Mailchimp) to communicate with learners, with automation software (Zapier) passing learner data to Mailchimp.

### Content and assessment development

We followed a “backward course design” process [39] for content development. We defined the target audience as HWs in Nigeria, with a focus on those working in primary health care facilities. Notably, these facilities are not typically staffed by physicians [40]. Team members from Resolve to Save Lives, AFENET-Nigeria and the National Primary Health Care Development Agency (NPHCDA) of Nigeria developed and reviewed the course. We created a sequence of modules to ensure consistent coverage of all objectives and content, and then wrote the modules based on that sequence. The pre-/post-test included questions drawn from, or similar to, some questions covered in other modules, and learners could review correct answers after completing both the pre-test and the post-test. We adhered to WHO guidance for technical content, but also ensured that the content was consistent with NPHCDA guidance. Research indicates that simple content adaptations may promote local relevance of learning materials [41]. To increase relevance to the target audience we gave all characters in the vignettes names that are common in Nigeria. Multimedia (images and video) used in the course were open-access or developed in-house. Three medical doctors on the team (based in the United States, Rwanda and Nigeria) reviewed all content for accuracy and alignment with NPHCDA standards. Collectively, these three technical reviewers have decades of experience working in IPC.

### Evaluation of learner perceptions

The Net Promoter Score (NPS) question – “How likely is it that you would recommend this training to a friend or colleague?” – has been used to evaluate user experience across a wide variety of industries, including education [42] and health care [43]. We asked this as an optional question at the end of each module with an 11-point response scale, ranging from not at all likely (0) to extremely likely (10). Responses were binned into promoters (9 and 10), neutral (7 and 8) and detractors (6 and below). The NPS was calculated as the percentage of promoters minus the percentage of detractors [43].

### Learner recruitment and communications

The training launched on April 6, 2021 and was available through June 1, 2021, a period of 8 weeks. We shared the link to the training through several Nigeria-based networks of HWs that we were part of, including WhatsApp groups and email lists. We also disseminated the training link via partner organizations’ social media platforms (Twitter and Facebook) and through an IPC bulletin that was distributed to various networks of HWs in Nigeria. Although the training was designed specifically for HWs and allied professionals, no restrictions were placed on participation (any person with the link could participate). Learners were allowed to participate anonymously, or they could consent to provide their name and email address in each module to work towards a certificate of participation. The requirement to complete all 10 modules was made clear to all participants on the course landing page and in other course materials. Participants received a confirmation email upon completing each module, and we emailed certificates to learners who completed all 10 modules (those who did not complete all modules did not receive certificates). In order to complete each module, learners had to answer all of the questions in the module. In modules 2-9, after multiple choice questions, learners received feedback as to whether or not they selected the correct response and an explanation of the correct response; they had the option to repeat questions they answered incorrectly. We did not require learners to repeat questions, and there was no required pass rate for the modules. We sent weekly email reminders to learners who had not completed the training, using Mailchimp to automate these reminders and customize them based upon course progression. Learner support was provided via email, with responses to support questions typically provided within 24 h.

### Data collection and analysis

We collected all data in SurveyMonkey and module completion was automatically recorded in Mailchimp. Invalid emails and duplicate enrollments were cleaned in Mailchimp and not included in enrollment and certification statistics. We analyzed all other data in Microsoft Excel and R version 3.4.4. If a learner completed the same module more than once, we only considered the first response to any given module. To enroll and work towards a certificate, learners had to complete a module and submit a valid email address with that module. Some respondents did not consent to share an email, but they could complete the modules anonymously for informational purposes. As a result, these responses were not identifiable and we excluded them from the analysis. To minimize survey fatigue, we kept the number of demographic questions to a minimum, focusing only on country and job role. Learners indicated whether or not they lived in Nigeria and selected their job role from a list; they could select more than one role and write in a role under “Other.” We coded these “Other” responses based upon keywords and key phrases. We evaluated learning gains among those who completed the pre-test and post-test in sequence (six learners who completed the post-test before the pre-test were excluded from the learning gains analyses). Each question on the pre-/post-test received equal weight, with a maximum score of one and a minimum score of zero. Partial credit was awarded for multiple answer (checkboxes) questions. We conducted qualitative analysis of a pre-/post-test question designed to elicit multiple strategies for IPC improvement by coding responses according to the WHO multimodal strategies framework [7]. Two individuals independently coded the responses, scoring each strategy as “mentioned” or “not mentioned.” The inter-rater reliability (Cohen’s kappa) was 0.83. In cases of discrepancies between the two coders, the given strategy was scored as “not mentioned.” To summarize support requests, we manually reviewed all emails we received from learners enrolled in the course from the course start date to 8 weeks after the course close date.

Pre-test scores and learning gains were not normally distributed (Shapiro-Wilk test), so we used the nonparametric t-test equivalents (the Wilcoxon rank-sum test and Wilcoxon signed-rank test) to compute inferential statistics for pre-/post-test scores. We used Cohen’s *d* to calculate effect sizes and chi-square tests to evaluate differences in the number of strategies named in open response questions. This project received a “non-human subjects research” determination from an IRB in the United States and an “exempt” determination from an IRB in Nigeria.

## Results

### Learner roles

All participants who responded to demographic questions reported living in Nigeria. Learners could select more than one role and an open response “Other” option was also available. Learners held a wide array of roles in the health system, including doctors, nurses, administrators, Ministry of Health officials and non-governmental organization (NGO) employees (Table 2). Common open responses in the “Other” category included terms such as “community health worker,” “medical laboratory scientist,” “environmental health officer” and “routine immunization officer.” Collectively, these results show that the training reached an array of professionals from across the health system in positions that require different levels of formal education and knowledge of IPC.

**Table 2:** Learner roles. Learners could self-identify with more than one role in a multiple-response question that included an open response “Other” field. “Other” responses (indicated with an asterisk*) were coded and grouped according to key words. We also manually coded and grouped “Other” responses that could not reasonably be attributed to health-sector roles (e.g., “administrative assistant,” “student” and “self-employed”)

Response	Percentage of total respondents
Health facility administrator or manager	15.5
Community health worker*	13.5
NGO employee	12.4
Ministry of Health official	12.1
IPC focal person	10.4
Nurse	9.3
Doctor or medical officer	9.0
Immunization officer*	5.4
Laboratory worker*	4.8
Environmental health worker*	3.7
Midwife	3.1
Hospital administrator or manager	3.1
Health facility maintenance staff or cleaning staff	2.5
Other public health*	2.5
Other role not health-related*	2.3
Head nurse or nurse manager	1.7

### Course usage

Three hundred seventy-two learners enrolled in the training, and of these, 220 (59%) completed all 10 modules and earned a certificate. Most attrition occurred early in the course (Table 3). The mean number of modules completed per learner was 6.8, and the mean delay between completion of the pre-test and post-test was 5.8 days. In this training, learners could skip modules, so some completed many modules, but did not earn a certificate. The median time to complete each module ranged from 8.5 min to 18.6 min, and the median time on task to complete all 10 modules was 138.6 min, or just under 2.5 h. Fourteen learners (4% of those enrolled) made a total of 18 support requests. The most common support requests (eight total) were related to certificates (e.g., asking when certificates would be sent, requesting that a certificate be re-sent or requesting a certificate in a different file format).

**Table 3:** Module topics and completion rates. The main topical focus of each module is given, along with the percentage of enrolled learners who completed that module. Certain themes, such as standard precautions and administrative controls, were integrated into many different modules. Learners could skip any module so there was no module that all enrolled learners completed

Module number and topical focus	Completion rate (%)
1 - Pre-test	95
2 - Hand hygiene	73
3 - Screening areas and practices	67
4 - Environmental cleaning	65
5 - Personal protective equipment	65
6 - Sharps safety	64
7 - Respiratory hygiene	62
8 - Transmission-based precautions	62
9 - Transmission-based precautions	62
10 - Post-test	63

### IPC knowledge and learning gains

The first and final modules of the course included a pre-test and post-test, respectively. The average pre-test score was 29% among the learners who completed the pre-test before the post-test (*N* = 222). Within the intervening modules (2-9), when learners answered formative assessment questions incorrectly, they received feedback on their answers and had the opportunity to try questions again. Learners provided a correct initial response to multiple-choice formative assessment questions 46% of the time. When learners answered incorrectly on their first attempt, they chose to repeat the question 78% of the time.

**Fig. 2:**
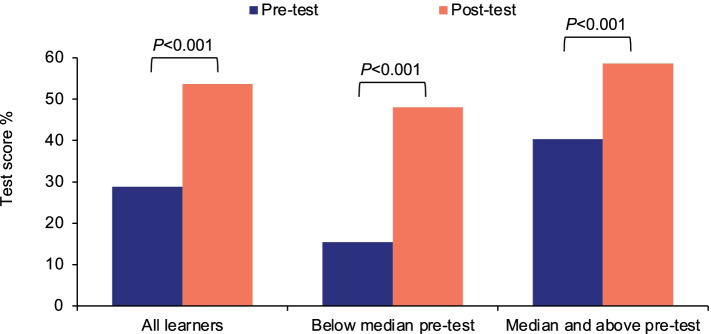
Pre-/post-test performance. Learners who completed both the pre- and post-test were grouped by pre-test score. Learners who scored below the median and at or above the median both had significant learning gains

Learners spent less time on the post-test (median of 9.9 min) than on the pre-test (median of 18.6 min). However, average post-test scores (Fig. 2) improved to 54%, representing a learning gain of 86% (*P* < 0.001) with an effect size of 1.22 (95% confidence interval: 1.00–1.44). This indicates that scores improved by more than one standard deviation from the pre-test to the post-test, a large effect size for an educational intervention [44]. We analyzed the post-test scores of those who scored below the median or at the median and above on the pre-test. Both groups demonstrated substantial and significant improvements. Moreover, there was no significant difference (*P* = 0.51) between the pre-test scores of learners who completed the post-test (mean pre-test score: 29%) and those who did not complete the post-test (mean pre-test score: 27%).

We also analyzed pre-test scores, post-test scores and learning gains by learner role, focusing on the most commonly reported roles (those with > 5% of enrolled learners). All of the groups analyzed had significant learning gains (Table 4).

**Table 4:** Pre- and post-test scores for the most common learner roles. Post-test scores were significantly different from pre-test scores in all of the groups (**P < 0.001; *P < 0.01)

Role	Pre-test %	Post-test %
Other - community health worker	30	52**
Doctor or medical officer	31	67**
Nurse	32	60**
IPC focal person	29	57**
Health facility administrator or manager	23	40**
NGO employee	29	53**
Ministry of Health official	35	60**
Other - immunization officer	28	50*

There was one open-response question in the pre-/post-test, in which learners were asked to name different facility-level approaches that could be taken to improve IPC. The responses were coded manually as described in Methods. Only 14% of learners mentioned two or more strategies on the pre-test; this increased to 25% on the post-test (*P <* 0.005).

### Learner feedback

We asked the NPS question (“How likely is it that you would recommend this training to a friend or colleague?”) at the end of modules 2-9 (all modules excluding the pre-/post-test). The mean rating was 8.95 out of 10, corresponding to an NPS of + 62, with a response rate of 46%.

## Discussion

We developed a new approach to training HWs in Nigeria that directly addresses the challenges they face in accessing online learning in general and IPC training in particular. The course reached hundreds of HWs who held diverse roles across the health system, with a high completion rate, positive feedback and significant learning gains. This demonstrates an effective and safe approach to train frontline HWs at scale in the context of COVID-19-related travel restrictions and safety measures.

### Low baseline knowledge of IPC

Low average scores on the pre-test and on learners’ first attempts of formative assessment questions suggest that this population had low baseline knowledge of IPC. In light of the recommendation that *all* HWs receive IPC training [7, 8], this indicates that more work needs to be done to build HW knowledge of foundational IPC concepts. The field would benefit from standardized IPC curricula and assessments that could be adapted to different country contexts and different levels of the health system [3]. Just as international standards endorsed by normative bodies contributed to quality improvement in medical education [45], a certification mechanism (for individual HWs, health facilities or both) would help policymakers identify gaps in IPC and develop targeted solutions. Moreover, the field would benefit from standardized monitoring and evaluation tools that would complement improved training programs.

### Completion and learning outcomes

The course had a high completion rate relative to industry standards. We are not aware of many reports of completion rates in optional, free, time-bound, fully online courses focused on HWs in Africa. One course, offered to HWs worldwide, had a reported completion rate of 36% [46]. There are many more reports on MOOC completion rates, which range from < 5 to 13% [17, 18], though rates are higher for some courses and platforms [14]. Some learners skipped modules or completed modules more than once; it would be useful to evaluate learners’ reasons for this in the future.

There was a substantial and statistically significant improvement in scores from the pre-test to the post-test, suggesting that learners’ knowledge of IPC increased by completing the course. Some learners completed the course quickly, but the learning gains we observed cannot reasonably be attributed to short-term recall of the questions themselves, as the average time elapsed between the pre-test and the post-test was about 6 days. Moreover, scores improved regardless of learners’ prior knowledge of IPC, and there was no significant difference in pre-test scores between those who did and did not complete the course. This is encouraging, given that low prior subject knowledge is associated with poor outcomes in other online courses [47]. Learners holding many different roles (e.g., doctors, nurses, administrators, community health workers) experienced learning gains in the course. Larger sample sizes and additional demographic information (including data on gender and education level) would facilitate a deeper understanding of differences in IPC knowledge among different groups of HWs.

### Learners’ perceptions of the course

The NPS of this course (+ 62) is slightly higher than NPS ratings of other self-paced free online learning experiences, such as Duolingo (+ 52) [48] and Coursera (+ 56) [42]. This indicates that learners were satisfied with the learning experience and were likely to recommend it to others (an NPS > + 50 is considered “excellent” [49]). The field of online learning lacks a consensus tool for rapid and standardized assessment of learner experience [42]; NPS may be a useful metric for comparing learner satisfaction across different online learning experiences and it would be even more helpful if widely adopted to allow for comparison against accepted benchmarks.

### Limitations

This training and its evaluation have some limitations. We did not address every challenge experienced by HWs in Africa who are trying to access online learning. This training required learners to have an internet connection and an email account and it was only offered in English. It remains to be seen whether this approach can effectively scale to train HWs in other countries, working in other levels of health care delivery and in other languages. We do not have a means of independently validating self-reported learner roles, and we do not have good a way to estimate how many learners saw the course. To improve the robustness and independence of learning gains evaluation, future courses should include pre-/post-tests that do not use questions that learners see in formative assessment. Moreover, we did not conduct long-term follow-up to assess retention of IPC knowledge, which is as important an outcome as short-term learning. We also did not assess behavior change, a downstream metric that is more challenging to capture, but also more relevant to important public health outcomes [50]. Though post-test scores improved, they remained low, which supports the concept that multiple reinforcing approaches are needed to sustain improvements in IPC knowledge and practice.

## Conclusions

This work demonstrates how organizations can take specific steps to understand their learners’ goals and barriers and develop solutions that build upon evidence from the science of learning. The barriers we identified to online learning among HWs in Nigeria are likely to be applicable in other contexts, and the solution we developed may be effective in different sectors and for other populations of learners. Moreover, this work suggests that immersion in formative assessment is an effective and enjoyable way for HWs to learn. Future work should evaluate long-term knowledge retention, the potential of the training to be scaled up to reach more HWs and the feasibility of implementation in other languages. The lessons learned in this pilot have informed a second phase of implementation of this course in multiple countries and in two languages. Our evaluation also points to foundational IPC knowledge gaps, suggesting a need for additional assessments and training for HWs in different roles within the health system.

## Data Availability

De-identified data are available from the corresponding author upon request.

## Abbreviations

HWs: Health workers
IPC: Infection prevention and control
MOOCs: Massive open online courses
NGO: Nongovernmental organization
NPHCDA: National Primary Health Care Development Agency
NPS: Net Promoter Score
WHO: World Health Organization
